# Supplementary material for: Identification of photoperiod-induced specific miRNAs in the adrenal glands of Sunite sheep (Ovis aries)
Source: Front Vet Sci. 2022 Jul 22;9:888207. doi: 10.3389/fvets.2022.888207 (PMC9354845; doi:10.3389/fvets.2022.888207)
Supplement: Supplementary file 1 [file Data_Sheet_1.ZIP › Supplementary file/miRDeep2 file.zip!/miRDeep2 file/Know_Structure/LP42A2/oar-mir-21.pdf]

miRBase precursor : oar-mir-21
Total read count : 219111
oar-miR-21 read count : 219093
remaining reads : 18

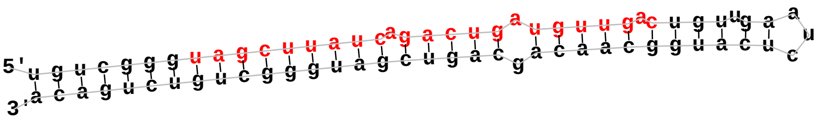

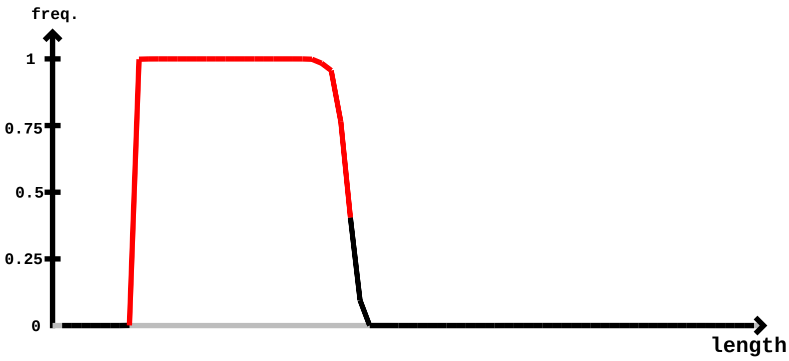

oar-miR-21

5'- ugucggguagcuuaucagacugauguugacuguugaaucucauggcaacagcagucgaugggcugucugaca -3'	exp		
((((((((((((((((.(((((.(((((.((((.((...))))))))))).))))))))))))))))))))	reads	mm	sample
......guagcuuaucagacugaug..	1	0	seq
......guagcuuaucagacugaugu...	2	0	seq
......guagcuuaucagacugauguu..	14	0	seq
......guagcuuaucagacugauguug...	67	0	seq
......guagcuuaucagacugauguuga..	35	0	seq
......guagcuuaucagacugauguugac...	13	0	seq
......guagcuuaucagacugauguugacu..	3	0	seq
.......uagcuuaAcagacugau...	1	1	seq
.......uagcuuaucagacugau...	17	0	seq
.......uagcuuaucagacugaug..	277	0	seq
.......uagcuuaucagacugaugu...	3241	0	seq
.......uagcuuaucagacugauguu..	5933	0	seq
.......uagcuuaucagacugauguug...	42016	0	seq
.......uagcuuaucagacugauguuga..	78703	0	seq
.......uagcuuaucagacugauguugac...	67707	0	seq
.......uagcuuaucagacugauguugacu..	20774	0	seq
.......uagcuuaucagacugauguugacug.......................................	4	0	seq
.......uagcuuaucagacugauguugacugu......................................	7	0	seq
.......uagcuuaucagacugauguugacuguu.....................................	9	0	seq
.......uagcuuaucagacugauguugacuguug....................................	2	0	seq
........agcuuaucagacugaugu...	5	0	seq
........agcuuaucagacugauguu..	7	0	seq
........agcuuaucagacugauguug...	38	0	seq
........agcuuaucagacugauguuga..	69	0	seq
........agcuuaucagacugauguugac...	76	0	seq
........agcuuaucagacugauguugacu..	26	0	seq
.........gcuuaucagacugauguu..	2	0	seq
.........gcuuaucagacugauguug...	11	0	seq
.........gcuuaucagacugauguuga..	9	0	seq
.........gcuuaucagacugauguugac...	10	0	seq
.........gcuuaucagacugauguugacu..	7	0	seq
..........cuuaucagacugauguuga..	1	0	seq
..........cuuaucagacugauguugac...	1	0	seq
...........uuaucagacugauguuga..	2	0	seq
...........uuaucagacugauguugac...	1	0	seq

oar-miR-21

```
ugucggguagcuuaucagacugauguugacuguugaaucucauggcaacagcagucgaugggcugucugaca
............uaucagacugauguugac..........................................    1    0    seq
............uaucagacugauguugacu.........................................    1    0    seq
.............................................caacagcagucgaugggcugu......    5    0    seq
.............................................caacagcagucgaugggcuguc.....    11   0    seq
.............................................caacagcagucgaugggcugucu....    2    0    seq
```
